# Using Blur for Perceptual Investigation and Training in Sport? A Clear Picture of the Evidence and Implications for Future Research

**DOI:** 10.3389/fpsyg.2021.752582

**Published:** 2022-03-02

**Authors:** Annabelle Limballe, Richard Kulpa, Simon Bennett

**Affiliations:** ^1^Univ Rennes, Inria, M2S-EA 7470, Rennes, France; ^2^School of Sport and Exercise Sciences, Liverpool John Moores University, Liverpool, United Kingdom

**Keywords:** blur, perceptual-cognitive skills, sport, perception, training

## Abstract

Dynamic, interactive sports require athletes to identify, pick-up and process relevant information in a very limited time, in order to then make an appropriate response. Perceptual-cognitive skills are, therefore, a key determinant of elite sporting performance. Recently, sport scientists have investigated ways to assess and train perceptual-cognitive skills, with one such method involving the use of blurred stimuli. Here, we describe the two main methods used to generate blur (i.e., dioptric and Gaussian) and then review the current findings in a sports context. Overall, it has been shown the use of blur can enhance performance and learning of sporting tasks in novice participants, especially when the blur is applied to peripheral stimuli. However, while intermediate and expert level participants are relatively impervious to the presence of blur, it remains to be determined if they are positive effects on learning. In a final section, we describe some of the methodological issues that limit the application of blur and then discuss the potential use of virtual reality to extend the current research base in sporting contexts.

## 1. Introduction

For more than two decades, sport scientists have investigated the contribution of perceptual-cognitive skills such as anticipation, attention, and decision making to expertise, and thereby expert performance (Mann et al., 2007b; Williams et al., 2011; Williams and Jackson, 2019). It is now well-accepted that experienced/skilled athletes in dynamic and interactive sports are better at anticipating an opponent's movement (Williams and Jackson, 2019) and consequently make earlier and more appropriate decisions (Raab et al., 2019). In addition, they can achieve high levels of performance on the basis of less information, whether that be due to temporal (e.g., information determined earlier) or spatial (e.g., information determined from fewer stimuli) constraints (Mann et al., 2007b; Williams et al., 2011; Williams and Jackson, 2019). Compared to novices, overall performance of experts is better when the environment is impoverished, thus indicating that the experts' ability to extract basic kinematic information is more resistant to visual stimuli deterioration. In part based on such findings, the role of visual functions such as acuity and contrast sensitivity have often been overlooked. Indeed, it is found in most sports that there is no additional advantage of supra-threshold levels of visual function (i.e., above the general population average), and thus little or no benefit of general visual training programmes aimed at improving vision to above-normal levels (Williams and Jackson, 2019). Somewhat counterintuitively, however, several studies have shown that perceptual and perceptual-motor behavior does not deteriorate (Mann et al., 2010a; Ryu et al., 2018; van Biemen et al., 2018), or can even be improved (Jackson et al., 2009; van Biemen et al., 2018), when the visual stimulus is artificially blurred by the experimenter. Early signs of an improvement of performance in the presence of blur (Jackson et al., 2009) has led to several studies on the use of blur to improve perception, anticipation, and decision making, with the largest improvements typically found in novices and/or less skilled participants (Jackson et al., 2009; Ryu et al., 2013, 2015, 2016). However, it seems there is less evidence for a positive impact of blur on performance of experts (van Biemen et al., 2018), and even a negative effect in some studies and particular tasks (Ryu et al., 2013, 2015, 2016).

In this article, we review and synthesize the current research on the use of blur in a sporting context, with an overall aim to provide some guidance for future research on perceptual training in sport. We include published articles that have manipulated blur in a sport setting/task using lenses (i.e., refractive blur) or image filtering (i.e., Gaussian blur). Accordingly, we begin by describing the potential impact of these different methods on the visual stimulus. We then consider the effect of blur on the perceptual-cognitive skills (i.e, anticipation and decision making) of novice and expert sports performers, followed by a section that describes the findings from perceptual-motor tasks where blur has been applied to the entire visual field or areas surrounding gaze location. We next consider if training in conditions of blur can facilitate learning and transfer, and finally we provide a critical reflection and discuss some perspectives for extending the applicability and scope of manipulating blur through the use of virtual reality.

## 2. Dioptric vs. Gaussian Blur – Conceptual Aspect

Normal healthy vision typically involves a seamless integration of high acuity and low acuity (i.e., blurred) inputs from the central and peripheral retina. Indeed, one does not normally notice that objects located away from the point of fixation are in fact blurred, even though this can provide useful information on spatial location (e.g., depth from crossed and uncrossed disparity). In normal or corrected-to-normal vision, high acuity input is perceived from cone cells that are concentrated at the center of the eye in the area known as the fovea. Cone cells detect light of different wavelengths, which is the basis of color perception, operate best in bright light, and enable perception of fine details (i.e., high spatial frequency). They thus play a critical role in being able to distinguish shapes and details of objects in our surrounds. The other main photoreceptor cells, known as rods, are not attuned to the same light wavelengths as cones, but they are more sensitive to light and are located mainly in the peripheral region outside the fovea. Although more numerous than cone cells, multiple rod cells converge onto single retinal ganglion cells, thus resulting in a specialization for processing low spatial frequency visual stimuli, as well as motion. Accordingly, in those with normal healthy vision, individuals fixate gaze location in a way to best combine high acuity and low acuity input. For example, in combat sports such as French boxing (Ripoll et al., 1995) and Karate (Williams and Elliott, 1999; Milazzo et al., 2016), experts direct their eye gaze mainly to the head and trunk of the opponent, whereas less experienced individuals tend to look at the opponent's fist and arms. Presumably, fixating gaze on a relatively static location (i.e., the center of the opponent's body) facilitates perception of information from central and peripheral vision. Indeed, minimizing the number of saccades to distal locations (i.e., opponent's fists and feet) would result in fewer periods of saccadic suppression, and thus a more continuous input of information^1^.

In the presence a partial central blur (e.g., macular degeneration), and hence the removal or alteration of high spatial frequency stimulus characteristics, individuals often respond by changing gaze fixation to a location that still enables a reasonable perception of object characteristics (Shima et al., 2010; Van der Stigchel et al., 2013). Similar effects on gaze location and outcome performance have been reported when a partial central blur has been artificially introduced by the experimenter (Ryu et al., 2015, 2016). Even when there is a full blur applied across central and peripheral vision, individuals can still exhibit high levels of performance in some tasks (Mann et al., 2010a) presumably from processing low spatial frequency input such as motion and spatial layout. Importantly, however, while the impact of both experimenter-imposed partial and full blur has been studied in a sporting context, this has been achieved using two different methods (i.e., dioptric blur and Gaussian blur). As we highlight below, these methods do not influence the stimulus in the same way and could thus impact upon the experimental findings.

A dioptric blur is equivalent to optical defocus caused by a visual impairment (e.g., myopia) or inaccurate accommodation. Experimentally, this is achieved using lenses (i.e., contact lenses or glasses) that alter focal length, thereby increasing focal length error and creating a *so-called* blur disk on the retina that is a function of defocus (in diopters) and pupil diameter. The consequence of wearing such lenses is a perturbation of high spatial frequency information, and thus a reduction in contrast sensitivity and visual acuity due to the stimulus edges being more difficult to discriminate (Watson and Ahumada, 2011; Kwon et al., 2016; Strasburger et al., 2018). The relative reduction in visual acuity (i.e., acuity with blur / best corrected acuity) can be derived as 1 / 1+D^2^, where D is the spherical error in diopters. Accordingly, as was reported by Bulson et al. (2008), it is possible to create different conditions of optical defocus, using convex and cylindrical lenses (PLANO, +0.50 D, +1.00 D, +1.50 D, +2.00 D, +1.00 D x 90, +2.00 D x 90, and +10 D) and estimate the corresponding level of visual acuity (20/20, 20/25, 20/60 20/100, 20/180, 20/25, 20/60, and 20/2000). Improving on this relative course estimation, Mann et al. (2010b) recognized the importance of taking into account best corrected visual acuity, although they did rely on the mean habitual visual acuity across participants (i.e., 6/5.3) when estimating the effect of three dioptric lenses (i.e., +1.00D, +2.00D, and +3.00D) on mean visual acuity (i.e., 6/11, 6/20, and 6/49).

The other experiments described in this review applied a Gaussian blur on images viewed on a computer monitor. A Gaussian blur is the result of an image or video processing procedure, whereby the optical system of the eye is unperturbed but the displayed stimuli are altered by removing the high frequency information (Watson and Ahumada, 2011; Kwon et al., 2016; Ma et al., 2018; Oleskiw et al., 2018; Strasburger et al., 2018). A Gaussian blur is different from dioptric blur in the sense that it does not create spurious resolution (see below) and does have a simple relationship with visual acuity. In terms of computation, the method requires the calculation of the transformation that needs to be applied to each pixel when rendering the image; it is the result of applying a Gaussian function to the pixel matrix. The Fourier transform decomposes the domains of frequencies contained in the image. Applying a Fourier transform to a Gaussian results in another Gaussian, and is thus similar to a low pass filtering to remove the high spatial frequencies (Ma et al., 2018; Strasburger et al., 2018). The new pixel coloration is the weighted average of the neighbored pixel characteristics included in the computation. Different software can be used to apply the processing, with directly implemented tool or custom processing. For example, Jackson et al. (2009) added 20% and 40% blur in both horizontal and vertical dimensions using the special effects module in Pinnacle editing software. Unfortunately, details on this method of applying the Gaussian were scant, thus making it difficult to compare both the experimental condition and results to other studies. van Biemen et al. (2018) also used software (Premiere Pro CC software) and the camera blur option to create their blur video, whereas Ryu et al. (2015, 2016) used the Adobe Premiere CS 4 software to apply a low-pass Gaussian filter with the 20, 50, and 100 units option.

**Figure 1:**
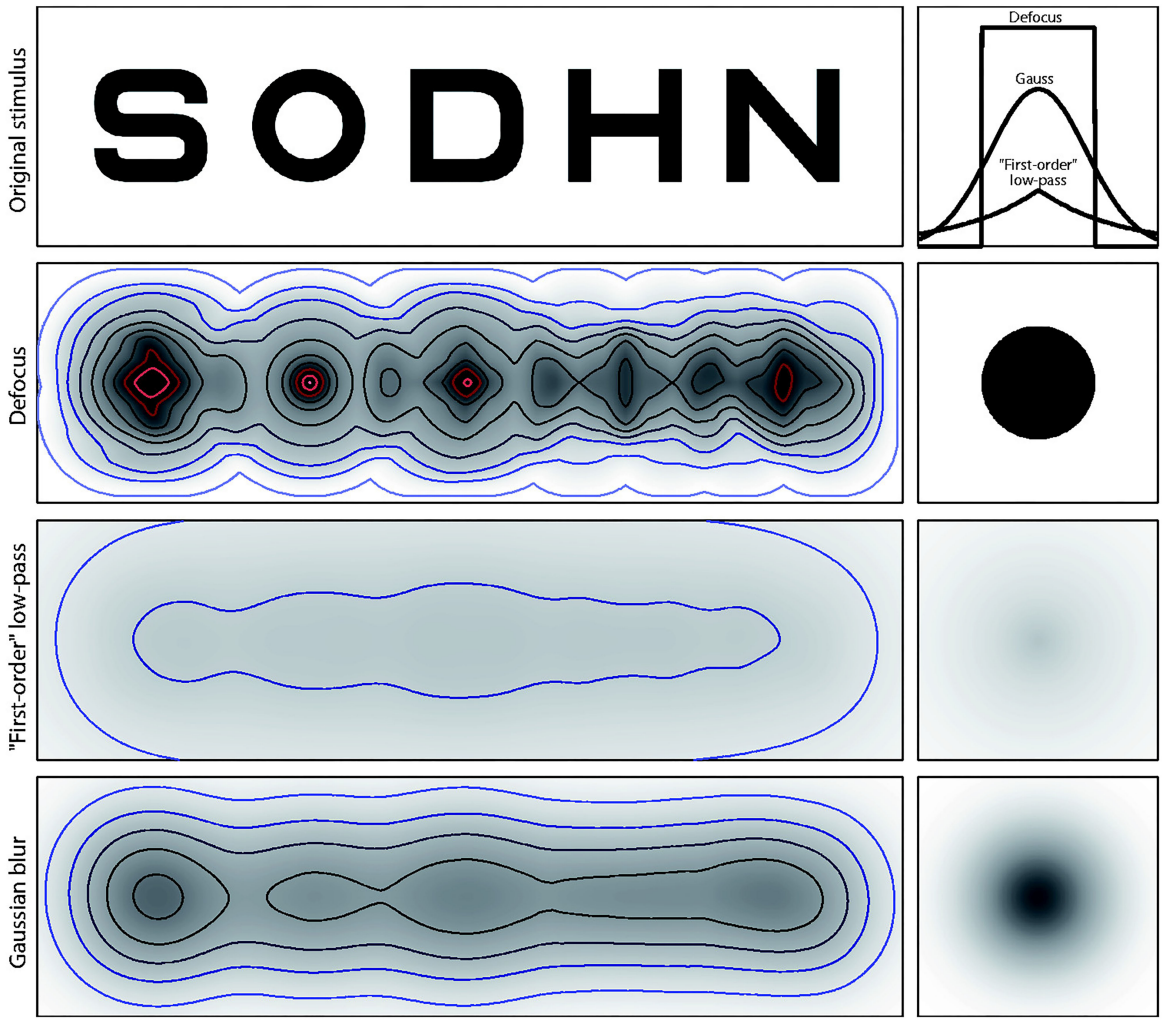
Illustration from Strasburger et al. (2018) of the effect of blur on Sloan letters. Top row: original, unblurred letters, together with point-spreadfunction profiles (right) for the lower rows. FWHMs of the three PSFs are equal. Note that PSF amplitudes are necessarily different since their volume needs to be normalized to unity (light is neither added nor lost). Second row: letters with dioptric blur simulated by using a disk with a diameter equal to the letter height as blur kernel. The effect of spurious resolution is so strong that the blurred letters look quite unlike their original. Third row: PSF with exponential drop-off (analogous to a first-order low-pass filter). Energy is spread over a wide spatial range, such that amplitude is rather low. Bottom row: letters with simulated Gaussian blur. For display, blurred images were increased in contrast to enhance the visibility of structures. Isolumes for all three patterns represent luminance steps of 7 percentage points (white 1/4 100%). The gray scale representation of the PSF in the right column uses a different scale than the blurred images (Strasburger et al., 2018) (CC BY 4.0).

It is often assumed that the dioptric and Gaussian blur are equivalent because they both alter the spatial frequencies of an image. However, as described by Strasburger et al. (2018), dioptric blur is a consequence of naturally occurring or experimenter-imposed alterations in physiological optics and is not identical to low-pass filtering (i.e., Gaussian blur) of a computer image. This becomes particularly evident when considering the point-spread function (PSF) that results from different methods of blur, and the resulting spurious resolution that is available in conditions of dioptric blur (see Figure 1). For example, Strasburger et al. showed that high spatial frequencies are detectable under dioptric blur, meaning that the orientation of a sine-wave grating may still be discernible but the appearance of blurred optotypes are likely too dissimilar from the original unblurred counterparts to permit identification without extended practice. No such spurious resolution is present when blur is created by low-pass Gaussian filtering (Strasburger et al., 2018). Consequently, while dioptric blur is easy to implement and is closest to the naturally-occurring blur caused by some visual impairments (e.g., myopia and presbyopia), it is debatable whether it is suitable for experimentation in a sporting context unless the study aims to investigate the effect of a visual impairment. That said, the application of Gaussian blur is not without limitations, such as the use of diverse software and computer algorithms, as well as a lack of coherence between the level of induced blur and visual acuity. These differences between dioptric and Gaussian blur have not been explicitly recognized in most research to date on blur in a sport context.

## 3. The Effect of Blur on Anticipation and Decision Making

Jackson et al. (2009) examined the influence of applying a low-pass Gaussian blur on configural information and anticipation during a dynamic perception task. Anticipation performance of tennis players (*N* = 32) with varying levels of competitive playing experience was examined using a 2-choice prediction task (i.e., judgement of direction). Video clips of a tennis serve occluded at 4 different times relative to final frame before ball contact (i.e., t1: -320 ms, t2: -160 ms, t3: 0 ms, t4: +160 ms) were presented on a computer screen, and viewed under 3 levels of blur (0% [no blur], 20%, and 40% blur). Based on performance in normal vision condition (i.e., 0%), participants were assigned to two groups: “good anticipators” and “poor anticipators.”

An interaction was found between anticipation skill (“good anticipators” vs. “poor anticipators”) and the level of blur. Performance of “good anticipators” was more disturbed with a low level of blur, such that performance declined significantly from the 0 to 20% blur condition (approximately -0.2 mean transformed accuracy). In contrast, performance of “poor anticipators” was slightly better in the 20% compared to 0% blur condition (approximately +0.03 mean transformed accuracy). Despite these significative group differences between the 0% and 20% blur conditions, performance of both groups was equal in the 20% blur condition. Moreover, both groups exhibited a similarly small increase in performance from the 20 to 40% blur condition. Overall, then, participants who were better at anticipating in the normal vision condition did not maintain this advantage with the introduction of the blur, as the authors expected. Only participants who were less competent in the normal vision condition exhibited an improvement in performance following the introduction of 20% and 40% blur, thus providing some preliminary support for the suggestion that the application of blur can make participants more attuned to basic kinematic information (i.e., motion).

According to the Jackson et al. (2009), an explanation for the non-linear effects of blur could be a change in participant's strategy. That is, in 20% blur condition, better anticipators could have still been looking for the high spatial frequency visual information, which was missing and thus performance declined. However, in 40% blur condition, participants may have changed strategy and thereby become more attuned to the essential movement kinematics that was present in the low spatial frequency information. While a reasonable account for the findings, the strategies used by players, whether unconscious or conscious, cannot be determined based on the recording of performance alone. Moreover, because there was only a single testing session, it is likely that blur acted as a momentary perturbation.

Based on the above findings, Ryu et al. (2018) designed a study using videos that showed either normal or blurred (i.e., low-pass Gaussian filter) badminton shots. Participants were 46 novices in badminton, who anticipated the direction of badminton shots across a pre-intervention/post study design. The pre-test and post-test comprised 96 video clips with different occlusion times (i.e., one frame before contact between racquet and shuttle, at the moment of contact, or one frame after contact) that showed deceptive actions. For the intervention, participants were divided into three training groups: low spatial frequencies only (i.e., blurred video), high spatial frequencies only (only detailed information conserved in the video) and normal vision. The frequency manipulations were made using low-pass and high-pass filters applied to the video images. The video clips showed non-deceptive action (*n* = 360) with different occlusion times, plus a direct performance feedback. The intervention lasted three days, with a total of 4 days from the pre-test to the post-test. A retention test was performed one week after the post-test. Eye movements were recorded in all sessions using an Eyelink II operating at 250 Hz.

The authors reported different effects on anticipation accuracy and reaction time when comparing the type of video training. When facing deceptive actions, the low frequency training group (i.e., blurred video), improved the most from pre-test to post-test, and then kept this advantage in the retention test one week later. This observation is consistent with the performance improvement of novices reported by (Jackson et al., 2009). The high frequency training group also improved from pre-test to post-test, and to a greater extent than the normal vision group. However, the advantage did not persist such that in the retention test, there was no difference compared to the normal vision (i.e., control) group. In addition, both groups performed worse than the low frequency group. When facing the non-deceptive trials, all groups exhibited a similar improvement in performance after training (response accuracy and reaction time). That is, the type of video (low frequency, high frequency, or normal) used in training did not influence performance while facing non deceptive trials.

In terms of gaze behavior, no changes were noted in fixation duration and breadth of search after training. A small change in saccadic amplitude was reported (*p* = 0.058), with larger saccades during retention test compared to pre-test and post-test, but this was the same for all groups. The time spent on the area of interest revealed some more notable differences between groups. The low frequency group (blurred video) spent more time with gaze located on racquet-shuttle contact in post-test and retention compared to normal group. However, they also spent less time fixating on the head compared to normal group but in the retention test only. The high frequency group spent less time fixating the head, compared to normal group but only in the post-test. There were no differences between these two groups at pre-test and retention test.

As described above, the trials used in training did not contain deceptive intent. Still, such training seemed to help participants recognize deceptive movement in post-test and retention, even though they did not face any examples of deceptive actions. According to the authors, the training intervention encouraged participants to rely more on the meaningful information, which they were then able to disambiguate from deceptive information in the post-test. This was supported by the finding that the low frequency training group decreased the time spent directing gaze toward the face of the opponent, instead locating gaze toward the racquet-shuttle contact, presumably to extract the most useful information. This pattern can be considered an improvement of visual strategy, and is consistent with the pattern of eye movement observed in experts (Mann et al., 2007a). Indeed, gaze direction and facial expression can be used to fake one's intention and mislead an opponent, thus indicating that the head area could be a source of distracting information (Petri et al., 2019). Conversely, the racquet-shuttle contact point contains important information about the direction of the shuttle and the type of shot.

The studies discussed up to this point were primarily based on novice populations (Applegate and Applegate, 1992; Bulson et al., 2008; Jackson et al., 2009; Ryu et al., 2018) and indicated some evidence that blur can improve anticipation performance, and potentially maintain this improvement following a relatively short, 7-day intervention (Ryu et al., 2018). These positive effects on performance and learning could have occurred because blur alters the process of visual information extraction. That is, applying a blur removes some irrelevant or even distractive information, which helps novices avoid an overload in visual processing when confronted with a multitude of stimuli. In the blur condition, the task could in effect become easier as participants are guided to the most useful information. However, such positive effects of manipulating blur are more limited for experts, and could even be detrimental. For example, it could be that experts have already learned to extract useful kinematic information from the environment, and as such removing high frequency information conveys no advantage, or even a disadvantage if experts use some aspect of high frequency information to support their perceptual-motor behavior. One such study of an expert population was reported by van Biemen et al. (2018), who investigated decision making of skilled football referees in a blurred condition. This represents an interesting population because although they are not skilled in performing the motor activity *per se*, referees are skilled in observing and detecting deceptive intent and movement of players. The 22 participants in this study were divided into two training groups: normal vision training and blurred vision training. The training took 45 min and was run the same day as a pre-test and post-test, which required the referees to make judgments on whether the situation shown in a video clip was a foul or not (26 clips, 13 foul, and 13 no foul). The training intervention comprised 70 clips in which all situations showed a foul. In this phase, the referees had to judge the severity of the foul, choosing to award a red card, yellow card, or no card as quickly as possible. Feedback on the correct response was given after each trial.

The authors found that the blurred vision group improved their response accuracy to a greater extent than the normal vision group, although this was not associated with a change in reaction time. The blurred vision group appeared to become more sensitive to genuine fouls, which echoed the results from Jackson et al. (2009) and Ryu et al. (2018) but this time with an expert population. However, at the individual level, some participants in the blur training group exhibited a decrease in performance. Although this was not discussed by the authors, one can suppose this could have been related to inattention or participant skill level. Therefore, although there is some evidence of a possible advantage afforded by blurring video images in training decision making of skilled participants, this requires further investigation. In addition, most of the above studies (Jackson et al., 2009) and (Ryu et al., 2018) used tasks where perception was not coupled to a motor response. This raises questions about the representativeness of the tasks compared to the actual sport setting and thus whether the findings from laboratory tasks are transferable.

## 4. Perceptual-Motor Behavior in the Presence of Blur

In an early investigation, Applegate and Applegate (1992) compared basketball shooting performance under five conditions in which dioptric blur was achieved using 6/6, 6/12, 6/24, 6/48, and 6/75 diopter lenses. The 19 male participants (all with a visual acuity of 6/6) performed 25 shots from one position in each condition. The authors observed a small drop in performance as a function of dioptric blur from 6/6 to 6/12. However, the decrease was not significant, and participants were still able to shoot the basket even with strong level of blur. Moreover, performance remained constant across all other levels of blur (from 6/12 to 6/75), thus showing that a static aiming task does not depend on high acuity information.

In another static aiming task (i.e., golf putting), Bulson et al. (2008) examined the effect of dioptric blur on 16 young participants. Similar to Applegate and Applegate (1992), participants performed the task while wearing different lenses: PLANO, +0.50 D, +1.00 D, +1.50 D, +2.00 D, +1.00 D, +2.00 D, and +10 D. These corresponded to visual acuity levels of approximately 20/20, 20/25, 20/60 20/100, 20/180, 20/25, 20/60, and 20/2000. It was found that the different levels of blur had little or no effect on completion of the golf putting task. The authors suggested that an adaptation to the blur could have explained the maintenance of performance. In addition, they suggested that automaticity related to repetition of practice at a fixed distance could have minimized the effect of a low level of blur. In fact, it is notable that in both (Applegate and Applegate, 1992; Bulson et al., 2008), the target was fixed and there was no need to consider information from moving teammates and/or opponents. Thus, it is questionable whether these findings generalize to dynamic sport situations where it is important to follow moving stimuli and anticipate future events.

To this end, Mann and colleagues conducted a series of studies using a cricket batting interception task, with intermediate to skilled cricket batters (Mann et al., 2007a, 2010a,b). In the first study Mann et al. (2007a), 11 intermediate batters faced a bowling machine under 5 conditions of refractive blur (PLANO, +1.00D, +2.00D, +3.00D, and normal correction lenses to control the effect of wearing lenses), with 4 different ball end locations. The authors confirmed that the performance of the cricket batting task did not require normal levels of visual acuity. In fact, there was even a tendency for better performance in +1.00D and +2.00D conditions compared to the PLANO condition. The only decrement in performance was found in +3.00D blur condition, which is recognized as the point of legal blindness.

**Figure 2:**
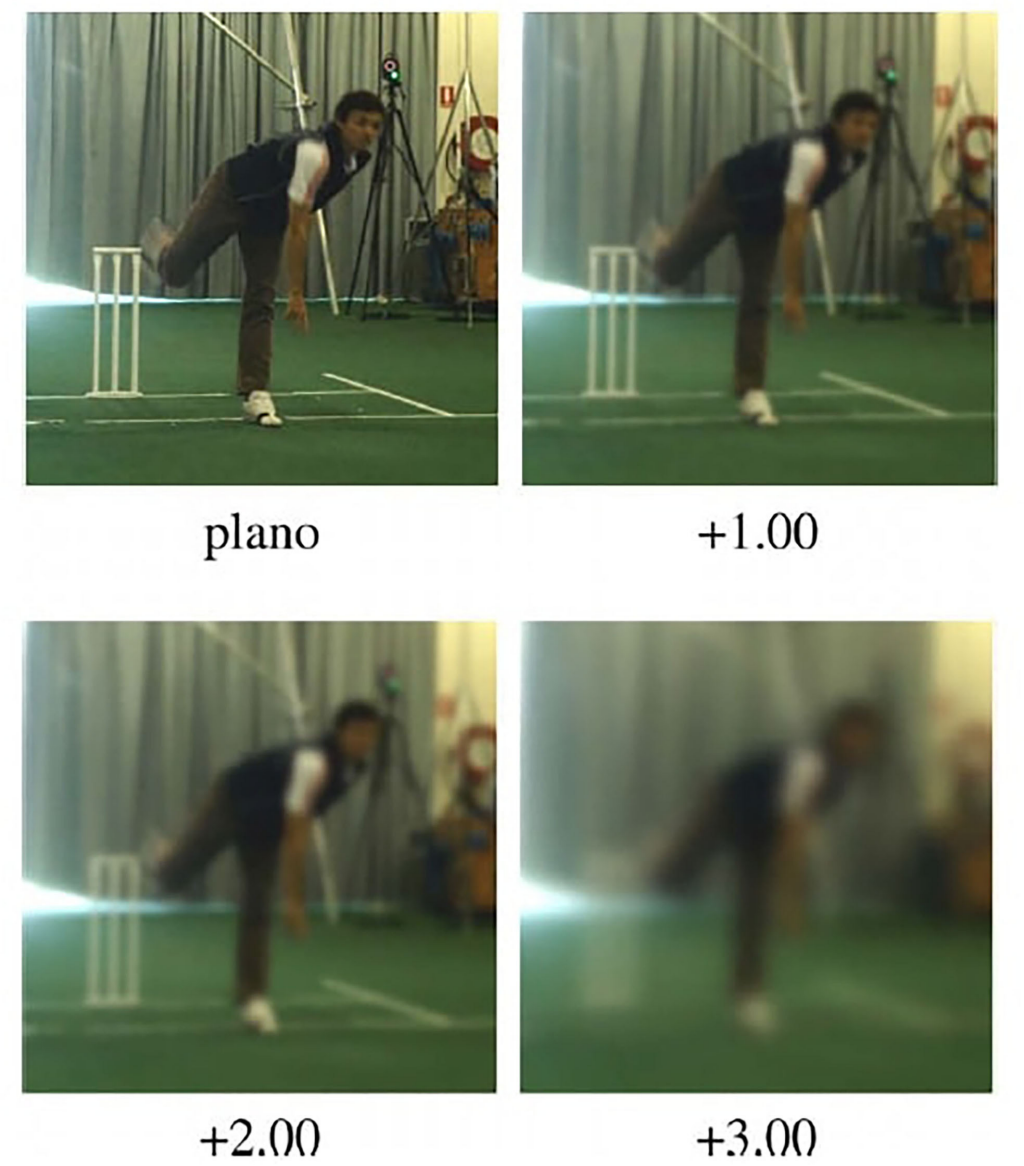
Illustration of Mann et al. (2010a)'s experiment: simulation of the four refractive blur conditions experienced by the participants. Reprinted from Mann et al. (2010a). Copyright 2010, with permission from Elsevier.

**Figure 3:**
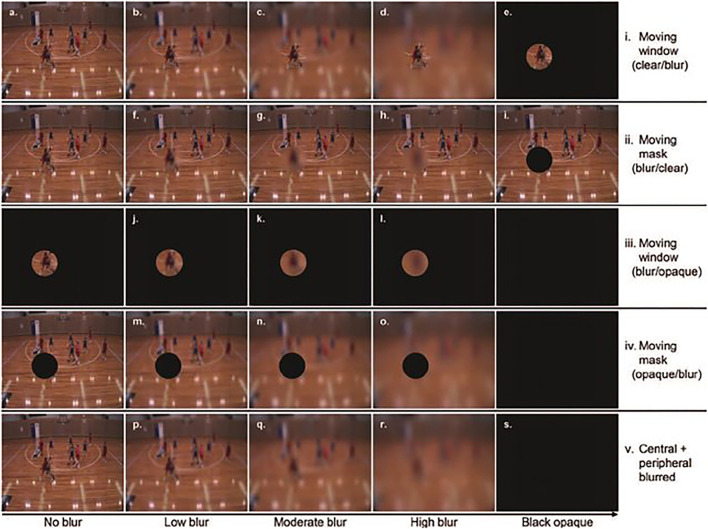
Screenshots of the 19 different viewing condition studied by Ryu et al. (2015): **(a)** full-clear, **(b–e)** moving window (clear/blur) conditions (low, moderate, high, and opaque, respectively), **(f–i)** moving mask (blur/clear) conditions (low, moderate, high, and opaque, respectively), **(j–l)** moving window (blur/opaque) conditions (low, moderate, and high, respectively), **(m–o)** moving mask (opaque/blur) conditions (low, moderate, and high, respectively); **(p–s)** central + peripheral blurred conditions (low, moderate, high, and opaque, respectively). The information in brackets (e.g., clear/blur) refers to the respective quality of the visual information in the central and peripheral sectors of the visual field. Reprinted from “The contributions of central and peripheral vision to expertise in basketball: how blur helps to provide a clearer picture” by Ryu et al. (2015). Copyright © 2015 by American Psychological Association. Reproduced with permission from Ryu et al. (2015).

In the second study, Mann et al. (2010a) compared the performance of 10 skilled male cricket batters when receiving balls delivered by a real bowler vs. a projection machine. Participants faced 196 trials, comprised of the 2 ball projection conditions, 4 blur conditions (PLANO, +1.00D, +2.00D, +3.00D, see Figure 2) and 3 different paced ball deliveries (medium-pace only for the machine, slow and fast-pace for the bowler). They found no differences in batting performance between the projection machine and real bowler, irrespective of the blur condition. Again, a significant decrease in batting performance was only found in the +3.00D blur condition. The authors suggested that the resilience of interception to refractive blur could be explained by the batting task being regulated by visual information processed in the dorsal stream, which is sensitive to motion and thus less affected by reduction in visual acuity. Moreover, they concluded that visual acuity is not a primary limiting factor in cricket batting and thus called into question whether there is benefit to improve visual acuity above an average level.

At this point, Mann and colleagues considered whether the effect of blur depended on the type of response required by the participant. To this end, Mann et al. (2010b), examined the influence of refractive blur in conditions where participants (10 skilled male cricket batters) responded to real ball deliveries by verbal response only (uncoupled) or verbal response plus batting movement (coupled). Two ball speed conditions were included (fast and slow), as well as 4 levels of blur (PLANO, +1.00D, +2.00D, +3.00D). In the uncoupled condition, performance (response accuracy) improved slightly from normal vision to the lowest level of blur, consistent with previous observations (Jackson et al., 2009; Ryu et al., 2018; van Biemen et al., 2018) and remained quite constant as the level of blur was increased. In the coupled condition, performance did not change from normal vision to the lowest level of blur and was better than the uncoupled condition. However, performance did deteriorate at the greater level of blur such that it was no better than performance in the uncoupled condition with the +2.00D blur manipulation, and somewhat worse than the uncoupled condition with the +3.00D blur manipulation.

Overall, then, the findings again indicate that anticipation performance can be improved (uncoupled condition) or maintained (coupled condition) in conditions of blur, and further that coupled anticipation is better than uncoupled anticipation (Farrow and Abernethy, 2003; Milner and Goodale, 2008; Van der Kamp et al., 2008). The authors suggested that the improvement in performance of the uncoupled task (fast-pace bowler) in some conditions of refractive blur could be explained by a shift in contribution from ventral to dorsal stream processing. A greater reliance on dorsal stream processing could also explain the maintenance of performance in the coupled condition. This interpretation is consistent with the notion that perceptual and perceptual-motor tasks are not dependent on a single processing stream and that the contribution from ventral and dorsal stream processing depends on the task demands and nature of the visual stimuli. For example, although in Mann et al. (2010b) there was no need to regulate a complex motor response when giving a verbal response, performance was influenced by the ability to anticipate motion of a fast moving visual object. Accordingly, while blurring the visual stimuli could impact negatively upon perceptual or perceptual-motor tasks that depend on normal levels of visual acuity to identify features and characteristics, this may not be the case if low spatial, high temporal frequency information such as object motion played a major role.

## 5. Peripheral and Central Blur

Many dynamic sporting tasks involve motion between oneself and surrounding objects, and thus require attention to be distributed successively and/or concurrently to different locations. Defined relative to gaze location, overt attention is involved in the processing of color and high acuity stimulus details in central vision, whereas covert attention is more involved in processing object location and motion in peripheral vision. Covert attention to information available in peripheral vision is suggested to play a major role in perceptual-cognitive expertise in dynamic sport settings (Hausegger et al., 2019; Vater et al., 2020). For example, novices tend to overtly attend to and process information in central vision, which means they often fail to perceive a large amount of information in the periphery. Experts, on the other hand, are better attuned to the relevant information, which they perceive simultaneously (overt and covert attention) in both central and peripheral vision.

As the perception of an object or limb as it moves through peripheral vision is based on processing of low spatial, high temporal frequency visual inputs, there should be minimal impact of applying a refractive blur to the entire visual field (Mann et al., 2007a, 2010a,b). In addition, it follows that blurring the entire visual field has limited ability to identify the contribution of specific sources and types of information that experts use to regulate perceptual-motor tasks in dynamic sport settings. This can potentially be overcome using a gaze-contingent blur manipulation (Ryu et al., 2013, 2015, 2016). The general idea is that because eye gaze does not necessarily coincide with covert attention, simply reporting spatial-temporal patterns eye gaze (i.e., visual search behavior) does not actually indicate what information is being extracted. Indeed, without additional manipulation, it can only be assumed that relevant information is picked-up in peripheral vision. With a gaze-contingent manipulation on a video display, the content of the video is adapted relative to gaze location, with an image artifact applied to a specific area of the display. For example, this method allows the experimenter to alter (e.g., occlude or blur) central vision whilst preserving normal peripheral vision, and *vice versa*.

To this end, Ryu et al. (2013) used an interesting approach to examine the role of central and peripheral visions, based on the gaze behavior, in a basketball decision-making task. Specifically, they used a gaze contingency display-change paradigm composed of 2 display conditions: a moving window or moving mask. In the moving window condition, only a circle of 5° surrounding the fixation point (i.e., gaze location) was visible, whereas in the moving mask condition, only information outside of a 5° circle surrounding the fixation point was available. In effect, the two conditions provided access to information in central vision (i.e., moving window) or peripheral vision (i.e., moving mask). The authors compared expert and novice participants in a full vision condition and the two gaze-contingent conditions. Video clips were presented showing sequences of a 5x5 basketball situation, where the participants viewed from a third person perspective and had to decide (i.e., button press response) the best action given the sequence presented (i.e., pass or drive to the basket). Overall, it was found that experts exhibited better decision-making performance in all conditions, thereby indicating a better capacity to use both central and peripheral visions. However, when discussing the limitations of their study, the authors pointed out that applying an opaque mask on the manipulated area could have changed a participant's gaze behavior. That is, by completely removing the visual information from some locations, participants may have shifted their gaze to another location in order to find sufficient information to make a correct decision.

To overcome the limitations of using an opaque mask, Ryu et al. (2015) revised their earlier 2013 protocol by applying a different level of blur to the gaze-contingent location. In total, there were 19 different viewing conditions (see Figure 3) comprised of 5 levels of blur (no blur, low, moderate, high blur, and opaque mask) and 5 configurations of visual alteration (see Figure 3, (i). Moving window—clear/blur. (ii). Moving mask—blur/clear. (iii). Moving window—blur/opaque. (iv). Moving mask—opaque blur. (v). Central + peripheral blur, complete blur). The moving window condition will be referred as peripheral blur and the moving mask as central blur. Only one aspect of vision was altered in conditions a to i (peripheral blur or central blur). In condition j to o, two aspects of vision were altered by combining the gaze-contingent blur manipulation with an opaque mask (e.g., condition j – low level central blur and opaque peripheral vision). In condition p to r, the combination of central and peripheral blur resulted in a uniform blur across the entire visual field. In experiment 4 of the paper, the video clips were occluded at a critical moment (i.e., when the ball carrier had to make a decision to which teammate to pass the ball). The participants (18 skilled and 18 less skilled male basketball players) were required to click on a vacant court (response slide) to indicate the position of the most appropriate teammate to receive the ball. Performance was measured in terms of response accuracy and response time.

Overall, in both the peripheral blur (clear central vision, blurred peripheral vision) and the central blur condition (blurred central vision, clear peripheral vision), performance of the experts was better than the novices for all levels of blur. When only one aspect of vision was altered, the experts performed above level of chance in all conditions. Noticeably, when information from a part of the visual field was unavailable (opaque central or opaque peripheral vision), experts were still able to perform above the level of chance. The authors suggested that experts were able to adapt and use information based on what was available. Findings for the novices indicated that they were unable to perform above the level of chance when central vision was blurred. However, it was interesting to note that novices did improve their response accuracy with moderate and high blur applied in peripheral vision (NB. there was no improvement for the experts in these conditions). This positive effect is in accordance with the previous results observed on novices (Jackson et al., 2009; Mann et al., 2010b; Ryu et al., 2018), although one should probably exert some caution when interpreting the facilitatory effect of blur because this was not evident when blur was applied to the whole field (c.f. Jackson et al., 2009; Mann et al., 2010b; Ryu et al., 2018).

In terms of the conditions where a part of vision was occluded by an opaque mask and the other part was blurred (see panel j. to o.), it was found that performance accuracy of experts was better than novices when a central blur (low and moderate blur) was applied (i.e., peripheral vision opaque). However, experts still performed better when there was no alteration of central vision. Performance of the novices was below the level of chance with blurred central vision and no peripheral vision. Moreover, when the opaque mask was applied to central vision, performance of experts was above the level of chance (contrary to novices), although it did decrease with the introduction of a peripheral blur. For both expert and novice participants, there was an increase in response time in the presence of blur (central or peripheral). Analysis of gaze behavior revealed somewhat mixed findings. Overall, fixation duration was affected to the same extent by central or peripheral blur when remaining visual field was occluded by an opaque mask, although there were some subtle differences for some levels of central blur. For example, fixation duration increased for the low and moderate central blur but decreased for the high level of central blur, all of which were still longer than the no-blur level. As for saccadic amplitude, both expert and novice participants exhibited an increase when there was a high blur applied to central vision and an opaque mask in the periphery. Finally, saccadic amplitude of novice participants decreased with the levels of blur.

## 6. From Performance to Learning

**Figure 4:**
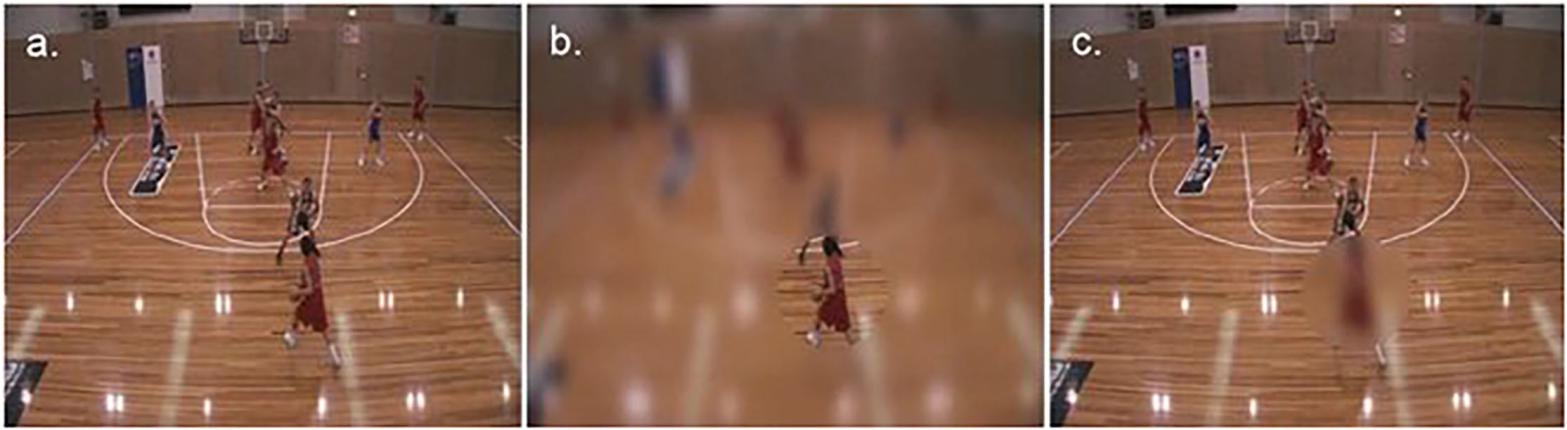
Illustration of Ryu et al. (2016)'s experiment: static screenshot of the **(a)** full-vision, **(b)** moving-window, and **(c)** moving-mask viewing scenarios. (CC-BY).

As described above, while intermediate and expert level participants are relatively impervious to the presence of blur, it seems that there is a facilitatory effect on performance of novices when blurring the entire visual field (Mann et al., 2007a) or only the periphery (Ryu et al., 2013, 2015). The next important question to be considered was whether these performance effects could enhance learning and ultimately be retained. To this end, Ryu et al. (2016) conducted a training study using the gaze contingent blur manipulation. Based on the materials and apparatus reported in Ryu et al. (2013, 2015), the previous study design was extended to include a pre-test (Day 1), intervention (Day 2–4), post-test (Day 5), and retention test (two weeks after the post-test). Fifty novice basketball players (recreational level) completed the pre, post, and retention tests in three different conditions (see Figure 4): full vision, peripheral blur, and central blur. The response was recorded by clicking on a vacant court slide to indicate the position of the most appropriate teammate to receive the ball. For the intervention, the population was divided into 4 groups that received different types of training: full vision, peripheral blur, central blur, and control group. Video clips taken from NBA gameplay were viewed in the respective training condition (occlusion at a key passing time), and participants had to decide as quickly and accurately as possible the most suitable action. Feedback on their response accuracy was provided after every trial. The exception was the control group, who watched an NBA dunk contest video, which did not involve any action play or decision-making.

The authors reported that in the retention test after training, the peripheral blur group (clear central vision, blurred peripheral vision) exhibited a slightly better improvement of performance (in terms of response accuracy) than the other 3 groups. They were also the only group to improve from pre-test to post-test, and then from post-test to the retention test in all viewing conditions. Conversely, performance of central blur group (blurred central vision, clear peripheral vision) decreased significantly from post-test to retention test, and almost back to the baseline, pre-test level. These results were observed across all the three viewing test conditions, that is, independently of the training modalities they received. Thus, training with the peripheral blur manipulation also led to an improvement in the full vision and central blur testing condition. Regarding eye behavior, the specific training regimen did not influence fixation duration. However, taking part in the training intervention per see (i.e, irrespective of training group), led to an overall increase of fixation duration. The authors suggested that the enhanced learning (i.e., best retention) following training in the peripheral blur condition was in part a result of the peripheral blur enhancing participants' use of information available in central vision. It was reasoned this occurred because the peripheral blur guided overt attention to information in central vision and away from potential distracting information in peripheral vision, thereby attenuating the overall attentional demands. Then, when transferring to the full vision condition, it was suggested that participants who had trained with a moving window were able to combine their newly learned enhanced use of central visual information with the unrestricted peripheral visual information now available, and thereby further improve decision-making performance.

More recently, Ryu et al. (2020) applied the gaze contingency manipulation to the detection of hazards in newly licensed drivers compared to experienced drivers. To better determine the mechanism involved in training using blur, the authors also measured cortical activity using EEG (i.e., alpha wave power). The logic is that an increase in alpha wave power reflects an increase in inhibitory control, whereas a decrease in alpha wave power suggests a neural activation. In this way, the high alpha wave power gives a view of basic cognitive processes, and in particular attention suppression and attention selection (Klimesch, 2012). Thus, in the situation of driving with blur, a decrease in alpha power would reflect a greater attention dedicated to visual processing (Klimesch, 2012; Ryu et al., 2020). In the first study of the experiment, performance of newly licensed drivers was compared to experienced drivers under full vision, central blur and peripheral blur conditions. Video clips were presented to participants that showed a non-adaptive first-person viewpoint. One or two hazards appeared in the visual field and participants had to identify them as quickly as possible by clicking on the video with a computer mouse. Overall, hazard discrimination and detection time were impaired in the central blur condition, while no differences were found between the other two conditions. The peripheral blur condition produced longer fixation duration and smaller saccadic amplitudes than the full vision and central blur conditions. Regarding the EEG findings, alpha wave power decreased in the peripheral blur condition and full viewing condition (e.g., more cortical activation). There was even some evidence that the peripheral blur condition offered the most benefit over some parameters like hazard processing time, with longer fixation on hazard and no difference in response time. According to the authors, the peripheral blur condition favored an efficient fixation leading to faster information processing, reflective of a visual search strategy that was more focused on central vision. This statement was supported by evidence of an increase of neural activation in cortical areas involved in perceptual-motor decision making information processing and motor response programming.

In the second study of the experiment, the authors conducted a training intervention on unlicensed trainee drivers. Based on the findings of the first study, the central blur condition was not included, so the design involved a comparison between a peripheral blur group (i.e., clear central vision and blurred peripheral vision) and a normal vision group. The study comprised a pre-test (Day 1), the training intervention (Day 2–5), post-test (Day 6), and a retention test one month after the post-test. The results indicated better performance of the peripheral blur group compared to the normal vision group, with the former exhibiting increased performance on hazard discrimination from pre-test to post-test, and post-test to retention test. Performance of the normal vision group remained similar across all tests. However, no change was found on hazard detection time, leading the authors to suggest that training influenced only spatial perception. In addition, it was found that neural activity (indicated by a decrease of the high alpha power) of the peripheral blur group was more important in retention test compared to the other group, leading to the suggestion that attention was more focused on relevant visual processing. For the normal vision group, neural activity decreased (indicated by an increase of the high alpha power) from post-test to retention test in parietal cortex, which is consistent with less integration of visual information.

Although encouraging, it should be remarked that a driving task involving hazard perception while passively observing a video display is quite different from real world driving, as well as a dynamic sport situation in which the relationship between the participant and surrounds is actively (not passively) modified by movement of the participant (e.g., whole-body or individual limb) and/or object of interest (e.g., opponent). It is perhaps not surprising, then, that occluding central vision during the driving task significantly impaired performance of both inexperienced and experienced drivers, whereas in the sport tasks studied to date experts are less impaired because they use both central and peripheral vision (Ryu et al., 2016; Hausegger et al., 2019; Vater et al., 2020). Still, despite these differences, the use of a peripheral blur for training the perception is encouraging. The described changes in neural activity also support this idea and give a better understanding of the effect of the blur manipulation. That is, an increase of cortical activity is associated with processing cues in central vision that are more closely aligned with the line-of-gaze (Ryu et al., 2020).

## 7. Critical Reflection

The primary aim of this article was to review the current research on how the artificial manipulation of blur influences perceptual-cognitive and perceptual-motor behavior in sport, and to what extent the application of blur during training can facilitate learning and transfer. We presented a narrative review, which although sometimes considered to be of lower status than a systematic review as it can contain bias (e.g., subjective selection of articles), was useful in this instance because it allowed us to bring together, summarize and critique articles from a new and hence small literature base. These articles included diverse methods of blurring (i.e., dioptric and gaussian blur applied to different tasks), different populations and varied experimental manipulations/questions. The narrative review allowed us to describe these differences in detail and to consider them in the wider context of how blur may be studied in future research using VR technology. As described in the previous sections, our review indicated that there are still unanswered questions regarding the underlying processing mechanisms that facilitate learning and transfer having been exposed to either a dioptric or gaussian blur while practicing a dynamic interceptive action. That aside, we agree with the authors that more work is required to better understand the effects of more prolonged training with a peripheral blur and if there is positive transfer from off-court training to on-court decision making performance. As we discuss below, and recognized by some of the authors of the studies described above, there are limitations of using pre-recorded 2D video clips with a non-adaptive 3rd person viewpoint. Similarly, although positive performance and learning effects of peripheral blur have been reported across a range of experimental tasks, some requiring no or a very limited motor response (i.e., button press), there are methodological issues that prevent the use of peripheral blur when training in more a natural sporting context. Part of the reason for the lack of work in such settings likely relates to the potential for injury that could occur if perception of an approaching object or person were disrupted. However, even if this were minimized (see Mann et al., 2010b), such training also does not lend itself to using a peripheral blur, which to date seems to be most effective in facilitating learning and transfer effects of novice participants (Ryu et al., 2016, 2020). Related to this point, it is notable that the positive effects of a gaze-contingent blur have yet to be shown in expert populations. So far, it has only been shown that experts are resilient to the introduction of blur to the entire visual field (Mann et al., 2010a; Ryu et al., 2015), and that a short period of training (i.e., 70 video clips) with such a manipulation can facilitate decision making accuracy in expert referees (van Biemen et al., 2018). However, there are many facets of expert performance, and it could be that some experts would benefit from training with blurred vision. For example, anecdotal reports from boxers and coaches suggest that during a fight, vision deteriorates with fatigue and repetitive blows to the head. Thus, it could be the case that boxers might benefit from safely practicing in a blurred vision condition if this encourages enhanced pick-up of information and/or provides an opportunity to familiarize to this specific situation. At the very least, the introduction of blur could be a new challenging situation that experts find stimulating, bringing a diversity to their training.

To overcome some of the limitations highlighted above, we are currently designing a methodology that uses virtual reality (VR) for training with a moving window in a more natural setting. According to Harris et al. (2019), “virtual reality is a collection of technologies that allow the user to interact with a simulation of some environment, in real-time, using their own senses and motor skills.” The strength of VR is that the constructed environment can be representative of the sporting context, but importantly for the purpose of research, it can be completely reproducible, controlled, and easily manipulated (Bideau et al., 2010; Williams et al., 2011; Miles et al., 2012; Craig, 2013; Stone et al., 2018; Faure et al., 2020; Harris et al., 2019; Renshaw et al., 2019). Unlike pre-recorded 2D video displays with a gaze-contingent blur (viewed from a 3rd person perspective), VR with integrated eye movement tracking (e.g., HTC Vive Eye Pro) ensures sufficient fidelity of binocular depth information in dynamic settings through viewpoint adaptation, while at the same time permitting a realistic, coupled motor response. This preservation of the normal perception-action loop means VR is not limited by the “standard one-size-fits all practice schedule” (Farrow, 2013; Stone et al., 2018; Renshaw et al., 2019). Instead, VR allows each individual to develop a particular interaction with the world and to experience it according to their skills, knowledge and morphology (Gibson, 1979; Fajen and Warren, 2007). In this way, VR offers the potential for individualized and targeted training that is respectful and adaptive to the level of the learner and the specific sporting context.

Using VR technology, we are studying the use of a gaze-contingent blur manipulation in boxing where the participant interacts with an opponent in a simulated fight setting. The technology developed allows the application of experimenter-determined Gaussian blur, and, will thus help us to identify the level of peripheral blur that facilitates learning and transfer when training with a peripheral blur. It can also help determine if the blur should be applied to the entire peripheral field (Ryu et al., 2016) or more specifically to areas of interest in the periphery that contains the relevant information. This could be relevant in combat sports such as boxing, where it is well-known that participants fixate gaze around the chest/head of the opponent and use peripheral vision to pick-up information from the hands and/or feet (Hausegger et al., 2019; Martínez de Quel and Bennett, 2019; Vater et al., 2020). Moreover, once the VR environment has been developed, it is relatively straightforward to manipulate parameters that impact upon task difficulty. This means that training can be adapted to the current and changing level of participants' performance, thereby providing a stimulating and challenging context throughout learning. This is a major advantage compared to pre-recorded 2D videos, where the stimuli cannot be individually manipulated or matched to a participant's skill level. Indeed, by continually challenging the participant, it should be possible to maintain motivation and facilitate the rate of learning (Michalski et al., 2019).

Integrating the blur manipulation within a VR environment could be promising and open new possibilities; however, some methodological and practical questions need to be considered. To reduce *so-called* cybersickness, and also enable depth perception similar to that experienced in the real world, it has been suggested that VR systems should ideally incorporate both depth of field (DoF) and foveated rendering techniques that introduce blur into the visual stimuli (Hussain et al., 2021). The former approach involves applying blur to visual stimuli that are located at different physical depths than the focal point. The applied blur is not uniform but follows a gradient such that it is stronger at more or less distant depths. The latter approach involves the application of blur in accord with the physiological properties of the retina (i.e., fovea, near periphery, mid-periphery); NB. Current HMDs do not typically enable presentation of objects in the far periphery. An object at the focal point that coincides with foveal vision is perceived with high acuity, whereas visual stimuli are increasingly blurred as they are located at more eccentric locations in the visual field.

Importantly, with dynamic, interactive VR stimuli, both DoF and foveated rendering require tracking of gaze location in order to optimally achieve the intended blur effect. The advent of eye tracking apparatus embedded within the HMD (e.g., Vive Pro Eye), has made this option more available. However, it does come at a cost in terms of purchasing the HMD and programming the VR stimuli. This could initially be problematic for practitioners and coaches but as the technology develops and interest grows, we envisage that low-cost, user-friendly solutions will be available. As for researchers, when applying a peripheral blur as suggested by the works of (Ryu et al., 2016, 2020), it will also be necessary to consider the potential interaction with DoF and foveated rendering. For example, the application of a high level of peripheral blur to a 3D VR stimuli could impact upon depth perception more than when applied to a 2D video image viewed from a third-person perspective.

## 8. Conclusion

In this review, we have considered how blurring all or parts of the visual information available to participants impacts upon their performance and learning of sporting tasks. Given the claims that elite athletes have better vision than novices and/or the general population, and that various aspects of vision can be improved through training, it may be surprising to find that athletes of varying skill level can maintain performance on a range of sporting tasks under relatively high levels of blur. Moreover, it is interesting to see that novice participants can even benefit from practicing with blurred vision, particularly when the blur is selectively applied to the peripheral visual field. That said, there are a number of methodological issues with current approaches to applying blur in a dynamic sporting context, as well as a lack of research on learning effects of training with blur learning in elite athletes. We suggest that the immersive and adaptive 1st-person perspective of virtual reality can overcome many of these limitations and thereby offers an opportunity for sports scientists to improve understanding of the benefits of training with blurred stmuli in sporting contexts.

## Author Contributions

AL, RK, and SB explored literature and wrote the manuscript, and review and edited the final manuscript. All authors approved the final version of this manuscript.

## Funding

This study was supported by the ANR within the framework of the PIA EUR DIGISPORT project (ANR-18-EURE-0022).

## Conflict of Interest

The authors declare that the research was conducted in the absence of any commercial or financial relationships that could be construed as a potential conflict of interest.

## Publisher's Note

All claims expressed in this article are solely those of the authors and do not necessarily represent those of their affiliated organizations, or those of the publisher, the editors and the reviewers. Any product that may be evaluated in this article, or claim that may be made by its manufacturer, is not guaranteed or endorsed by the publisher.

## Footnotes

^1^In addition, when gaze is fixated on an object at a particular depth, surrounding objects not located on the horopter will be perceived at different depths. Just beyond the horopter, disparity in the retinal images provides a reliable cue to depth. However, as the surrounding objects move further away from the point of gaze, blur can also provide an important contribution to depth perception (Held et al., 2012; Langer and Siciliano, 2015).
